# High Nutrient Transport and Cycling Potential Revealed in the Microbial Metagenome of Australian Sea Lion (*Neophoca cinerea*) Faeces

**DOI:** 10.1371/journal.pone.0036478

**Published:** 2012-05-11

**Authors:** Trish J. Lavery, Ben Roudnew, Justin Seymour, James G. Mitchell, Thomas Jeffries

**Affiliations:** 1 School of Biological Sciences, Flinders University, Adelaide, South Australia, Australia; 2 Plant Functional Biology and Climate Change Cluster (C3), University of Technology Sydney, Broadway, New South Wales, Australia; Biodiversity Insitute of Ontario - University of Guelph, Canada

## Abstract

Metagenomic analysis was used to examine the taxonomic diversity and metabolic potential of an Australian sea lion (*Neophoca cinerea*) gut microbiome. Bacteria comprised 98% of classifiable sequences and of these matches to *Firmicutes* (80%) were dominant, with *Proteobacteria* and *Actinobacteria* representing 8% and 2% of matches respectively. The relative proportion of *Firmicutes* (80%) to *Bacteriodetes* (2%) is similar to that in previous studies of obese humans and obese mice, suggesting the gut microbiome may confer a predisposition towards the excess body fat that is needed for thermoregulation within the cold oceanic habitats foraged by Australian sea lions. Core metabolic functions, including carbohydrate utilisation (14%), protein metabolism (9%) and DNA metabolism (7%) dominated the metagenome, but in comparison to human and fish gut microbiomes there was a significantly higher proportion of genes involved in phosphorus metabolism (2.4%) and iron scavenging mechanisms (1%). When sea lions defecate at sea, the relatively high nutrient metabolism potential of bacteria in their faeces may accelerate the dissolution of nutrients from faecal particles, enhancing their persistence in the euphotic zone where they are available to stimulate marine production.

## Introduction

Mammalian body surfaces are colonised by microbial communities that often exist in a mutualistic relationship with their mammalian host [1]. Mutualistic interactions between the gut microbiota and mammalian hosts have evolved over a long co-evolutionary process [2]. The microbial community of an organism is termed the ‘microbiome’ and the gastrointestinal microbiome has a crucial role in gut physiology, defence against pathogens, maturity of the immune system and the recovery of metabolic energy for the host [3]. The gut microbiome synthesises vitamins and amino acids and aids in the breakdown of otherwise indigestible foods [1].

Gut microbes have previously been examined by isolating and sequencing bacterial species from faeces [3]. However, the advent of metagenomic techniques has allowed for a more comprehensive and unbiased assessment of microbial genomic diversity within the complex gut ecosystem by allowing for examination of organisms not easily cultured in a laboratory [4]. Metagenomic analysis of faeces allows for characterisation of the microbial community within the gut [1] and can elucidate important processes for the gut microbes and the host and provide insight into links between the host, gut microbes and the surrounding ecosystem [1], [3]–[5].

Here we characterise the community composition of an Australian sea lion faecal microbiome and compare the metabolic potential with other microbiomes. In doing so, we provide the first information on the gut microbiome of an Australian sea lion. We examine a marine mammal specifically, in light of recent research highlighting the role of marine mammal faeces in the nutrient cycle of the ocean [6]. We consider whether bacteria might enhance the persistence of Australian sea lion faecal nutrients in the photic zone by solubilising nutrients from the faecal particles before the faecal particles can sink to the deep ocean.

## Methods

### Sample Collection

Australian sea lions (*Neophoca cinerea*) number approximately 11 000 with the major population occurring in South Australia [7]. Australian sea lions predominantly consume squid and fish prey and dive to average depths of roughly 40–80 m while foraging [8]. A faecal sample from an Australian sea lion was collected from Seal Bay, Kangaroo Island, South Australia (35°59.842′S, 137°19.484′E). The sample was collected within 20 minutes of defecation using a sterile scalpel and care was taken to ensure that sampling did not include any faeces in direct contact with the ground or contaminated by seawater. The sample was placed in sterile 50 ml plastic tubes and retained on ice at approximately 4°C for <12 hours during transport. The sample was then frozen at −80°C.

### Metagenomic Sequencing

Microbial community DNA was extracted from 30 grams of faeces using a bead beating and chemical lysis extraction kit (MoBio, Solano Beach, CA.) and further concentrated using ethanol precipitation. DNA quality and concentration was determined by agarose gel electrophoresis and a nanodrop spectrophotometer respectively. Over 6 µg of high molecular weight DNA was sequenced using a 454 GS FLX (Roche) pyrosequencing platform at the Australian Genome Research Facility.

### Data Analysis

Unassembled sequences were annotated using the MetaGenomics Rapid Annotation using Subsystem Technology (MG-RAST) pipeline version 2.0 (http://metagenomics.nmpdr.org/) [9]. The MG-RAST pipeline implements the automated BLASTX annotation of metagenomic sequencing reads against the SEED non-redundant database [10], a manually curated collection of genome project-derived genes grouped into specific metabolic processes termed ‘subsystems’. The SEED matches of Protein Encoding Groups (PEGs) derived from the sampled metagenome may be reconstructed in terms of either metabolic function of taxonomic identity at varying hierarchical levels of organisation. The MG-RAST pipeline was used to perform quality control on the sequences by removing reads with greater than 10 ambiguous bases per read and dereplicating artificial duplicates in which the first 50 bp of the read were identical. Phylogeny was assigned by matching sequences to the SEED database [10] using BLASTX with an e-value of 10^−5^ and a minimum alignment length of 50 bp. Similarly, sequence reads were assigned to metabolic subsystem pathways using MG-RAST and a BLASTX e-value cut-off of 10^−5^.

The metabolic potential of the Australian sea lion faecal microbiome was compared to metagenomes sequenced from other faecal samples, seawater samples and whale fall samples publicly available on the MG-RAST server using PRIMER. Relative proportions of metabolic subsystem categories were generated using the heatmap function in MG-RAST before being exported to PRIMER. Relative proportions were normalised by sequence matches to control for sequencing effort before being square root transformed. Bray Curtis similarity was used to construct a Multi-Dimensional Scaling plot. The MDS was used to determine the sample that most closely clustered to the metabolic potential of the Australian sea lion faecal microbiome. The STatistical Analysis of Metabolic Profiles (STAMP) package [11] was used conduct a Fisher’s exact test with the Storey’s FDR correction applied in order to conduct a fine scale examination of differences in metabolic potential between the Australian sea lion faecal microbiome and the most similar sample. Corrected P-values (q-values) were calculated with those that were <0.05 being deemed significant. The corrected p-value indicates the expected proportion of false positives within the set of features with a smaller q-value. A Fisher’s exact test was also carried out between the Australian sea lion faecal microbiome and a healthy fish gut microbiome to elucidate differences between organisms that share a similar environment. We then considered gene sequences that are over-represented in the Australian sea lion faecal microbiome compared to both the most similar metagenome and the fish gut microbiome and gene sequences that are over-represented in the Australian sea lion faecal microbiome compared to two Antarctic seawater samples. To facilitate comparison between metagenomes with smaller read lengths no minimum base pair alignment length was set when comparing microbiomes. The Australian sea lion faecal microbiome is publically available on the MG RAST pipeline (http://metagenomics.nmpdr.org/, MG RAST ID: 4446343.3).

## Results

### Australian Sea Lion Faecal Bacteria Taxonomy

Whole community microbial DNA from a fresh sample of Australian sea lion faeces was sequenced and yielded 45 760 contigs totalling 14 124 226 base pairs with an average fragment length of 309. A total of 20 843 sequences (45.55%) could be matched to proteins in SEED subsystems. Of these, 98% of similarities were to bacterial, 1.38% to archaea, 0.46% to eukaryota, 0.17% to viruses and 0.01% were to plasmids. Our data represents the most abundant members of the community which are thriving in the current ecological conditions and does not address the ‘rare biosphere’ of low abundance taxa. This is an inherent feature of all metagenomic studies and is adequate when inferring metabolic potential because a large amount of biogeochemical cycling is carried out by the most abundant community members.

**Figure 1:**
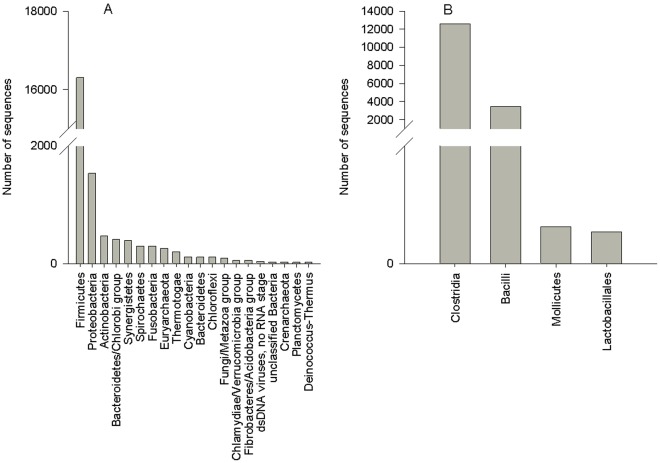
Taxonomic Diversity of Australian Sea Lion Gut Microbiome. A: The Australian sea lion gut microbiome was dominated by *Firmicutes* and *Proteobacteria*. The following phyla were also present in the ASL gut microbiome but had <10 sequences and thus are not shown on the graph: *Aquificae*, *Viridiplantae*, *Korarchaeota*, Bacteriophage ROSA, *Englenozoa*, *Lactobacillus plantarum* bacteriophage phiJL-1, Plasmid PCD4, Plasmid pIP404, Environmental samples, ssRNA negative strand viruses. B: *Firmicutes* were in turn dominated by *Clostridia* and *Bacilli*.

Bacterial phylogenetic diversity was dominated by *Firmicutes* (80% of bacterial sequences), *Proteobacteria* (8% of bacterial sequences) and *Actinobacteria* (2% of bacterial sequences) (Figure 1A). *Firmicutes* were dominated by *Clostridia* (77% of *Firmicutes*) and *Bacilli* (21% of *Firmicutes*) (Figure 1B). *Proteobacteria* were dominated by *Gammaproteobacteria* (49% of *Proteobacteria*) and Alphaproteobacteria (13% of Proteobacteria) (data not shown).

### Australian Sea Lion Faecal Bacteria Metabolic Analyses

**Figure 2:**
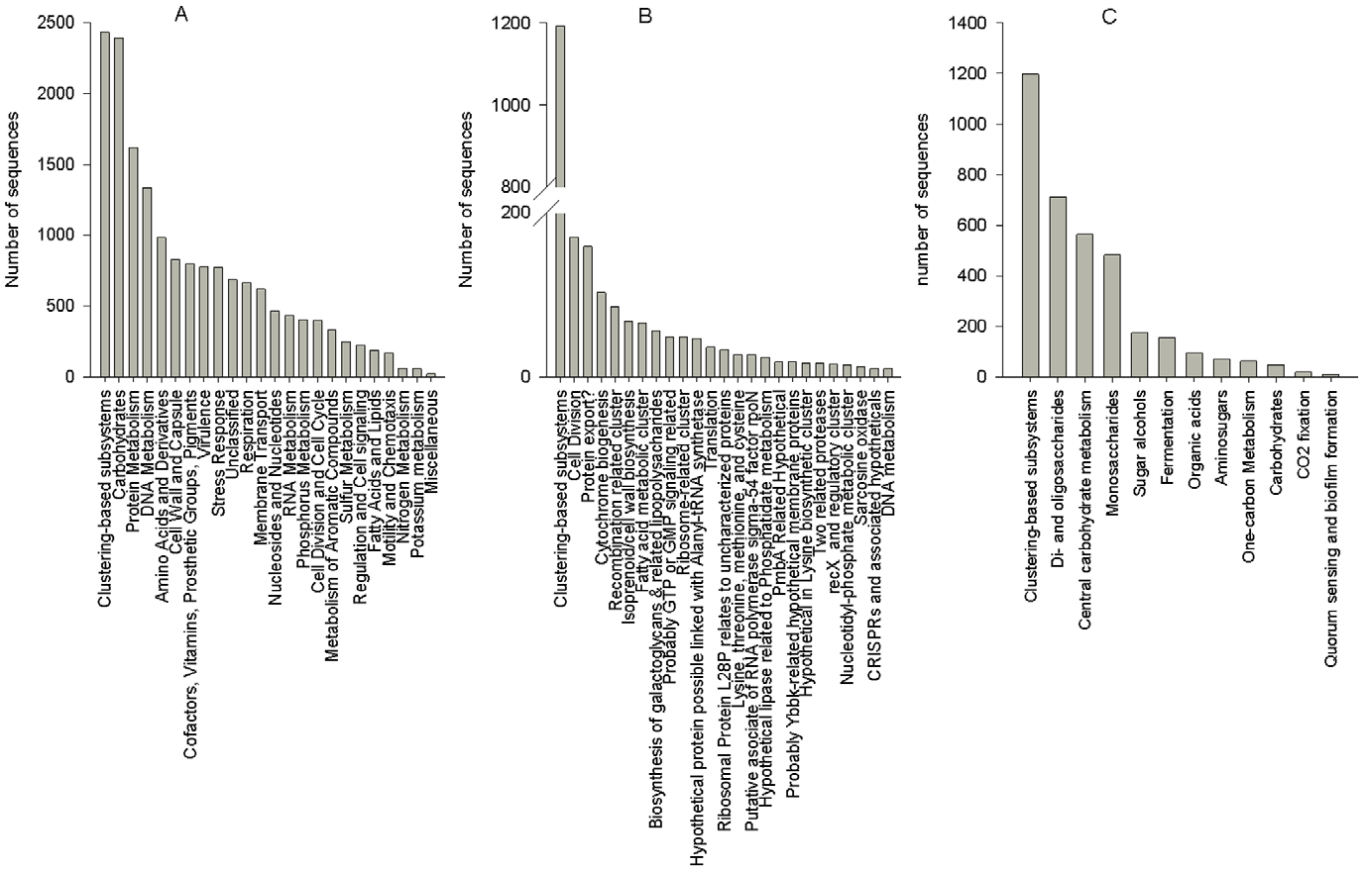
Metabolic Potential of Australian Sea Lion Gut Microbiome. A: The metabolic potential of the Australian sea lion gut microbiome is dominated by clustering-based subsystems and carbohydrates. Protein metabolism and DNA metabolism are also highly represented. Sequences coding for prophage, secondary metabolism, macromolecular synthesis and dormancy and sporulation were also present but were represented by <10 sequences each and hence are not shown here. B: The metabolic potential of the clustering based subsystems in the Australian sea lion gut microbiome are dominated by clustering based subsystems, cell division and protein export. The following metabolic functions were also present but had <10 sequences and are not shown here: hypothetical associated with RecF, carotenoid biosynthesis, tricarboxylate transporter, probably organic hydroperoxide resistance related hypothetical, protein, pigment biosynthesis, related to N-acetylglucosamine utilization subsystem, TldD cluster, tRNA sulfuration, chemotaxis, response regulators, cluster of unknown function, DNA polymerase III episolon cluster, lipoprotein B cluster, putrescine/GABA utilization cluster, D-tyrosyl-tRNA (Tyr) deacylase (EC′3.1.-.-) cluster, metaylamine utilisation, putative GGDEF doman protein related to agglutinin secretion, and siderophore biosynthesis. C: The clustering-based subsystems were further dominated by clustering-based systems (hierarchical level 3), di- and oligosaccharides, central carbohydrate metabolism, monosaccharides.

The metabolic potential of the Australian sea lion faecal microbiome was dominated by a clustering based subsystem (14%) and genes coding for core metabolic functions such as carbohydrate utilisation (14%), protein metabolism (10%) and DNA metabolism (7%) (Figure 2A). The clustering based subsystem was in turn made up of a clustering subsystem category (43%) which included putative hemin transporters and bacterial RNA metabolizing Zn dependent hydrolases (data not shown), 6% cell division and 6% protein export (Figure 2B). Carbohydrate utilisation (Figure 2A) was made up of 33% clustering based subsystems, 20% di- and oligosaccharides and 15% central carbohydrate metabolism (Figure 2C).

### Comparison of Australian Sea Lion Faecal Microbiome with other Faecal, Seawater and Whale Fall Microbiomes

**Figure 3:**
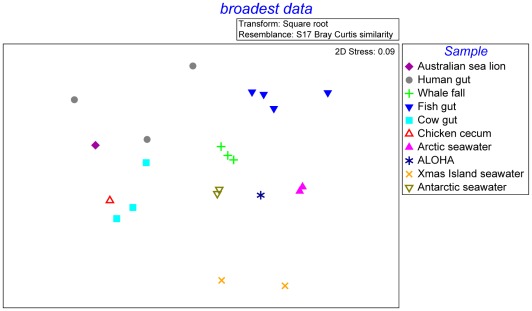
Multi-Dimensional Scaling Plot Comparing Australian Sea Lion Microbiome Metabolic Potential with several other Gut, Seawater and Whale Fall Microbiomes. Metabolic potential of the Australian sea lion gut microbiome is compared to publicly available seawater samples (Antarctic, North Pacific, South Pacific and Indian Oceans), gut microbiomes (human, fish, cow and chicken), and whale fall microbiomes from the MG-RAST server.

The metabolic potential of the Australian sea lion faecal microbiome was compared to 21 microbiomes publicly available on the MG-RAST server. The compared microbiomes comprised of seawater samples (Antarctic, North Pacific, South Pacific and Indian Oceans), gut microbiomes (human, fish, cow and chicken), and whale falls (Table 1). The Australian sea lion faecal microbiome clustered most closely with human gut microbiomes, with avian and cattle gut microbiomes also clustering near the sea lion faecal microbiome (Figure 3).

**Table 1:** Publically Available Metagenomes used for Comparison with the Australian Sea Lion Gut Microbiome. Number of hits determined with BLASTX E value of 10^−5^, no minimum base pair alignment length.

Title	MG-RAST ID	Description	Number of hits (phylogeny)	Number of hits (metabolism)
Sea lion	4446343.3	Australian sea lion faeces	24297	16804
Human(A)	4440946.3	Human faeces - Kurokawa human In-A	16743	11967
Human(B)	4440945.3	Human faeces - Kurokawa human In-B	8801	5306
Human (C)	4440940.3	Human faeces – Kurokawa human F1-U	14896	12275
Cow(A)	4441679.3	Cow rumen –640F6	24443	16189
Cow(B)	4441682.3	Cow rumen – pooled plankton	24600	15745
Cow (C)	4448367.3	Cattle faecal pool	156192	100945
Fish(A)	4441695.3	Fish – Healthy gut bacteria	12453	7544
Fish(B)	4441696.3	Fish – morbid gut bacteria	13307	8086
Fish (C)	4440066.3	Aquacultured fish	11667	7405
Fish (D)	4440065.3	Aquacultured fish	5237	3263
Chicken	4440283.3	Chicken cecum	54877	30674
Antarctic (A)	4443686.3	Antarctica Aquatic Microbial Metagenome_8	92148	69892
Antarctic (B)	4443687.3	Antarctica Aquatic Microbial Metagenome_9	89222	68848
Arctic (A)	4440306.3	Arctic seawater	81674	52807
Arctic (B)	4441622.3	Arctic seawater – Chukchi	135541	75370
Xmas (A)	4440038.3	Northern Line Islands	45741	33654
Xmas (B)	4440041.3	Northern Line Islands	5484	2740
ALOHA	4441057.4	HOT/ALOHA upper euphotic	6590	4426
Whale fall	4441619.3	Whale fall bone	36057	25884
Whale fall	4441656.4	Whale fall mat	32133	23177
Whale fall	4441620.3	Whale fall rib	34525	26119

The human gut microbiome (termed Human A) most similar to the sea lion faecal microbiome was used for a finer scale examination of the differences in taxonomic and metabolic potential between the two samples. Statistical analyses revealed a total of 23 significant differences in taxonomic diversity between the Australian sea lion faecal microbiome and the Human A faecal microbiome (Figure S1). The Australian sea lion faecal microbiome was over-represented in *Firmicutes* and under-represented in *Bacteroidetes* compared to the Human A faecal microbiome. There were 63 significant differences in metabolic potential between the Human A and Australian sea lion microbiomes (Figure S2). The sea lion microbiome was over-represented in comparison to Human A microbiome in regard to 28 functions and pathways including electron accepting reactions, protein biosynthesis, ABC transporters, phosphorus metabolism and iron scavenging mechanisms.

**Figure 4:**
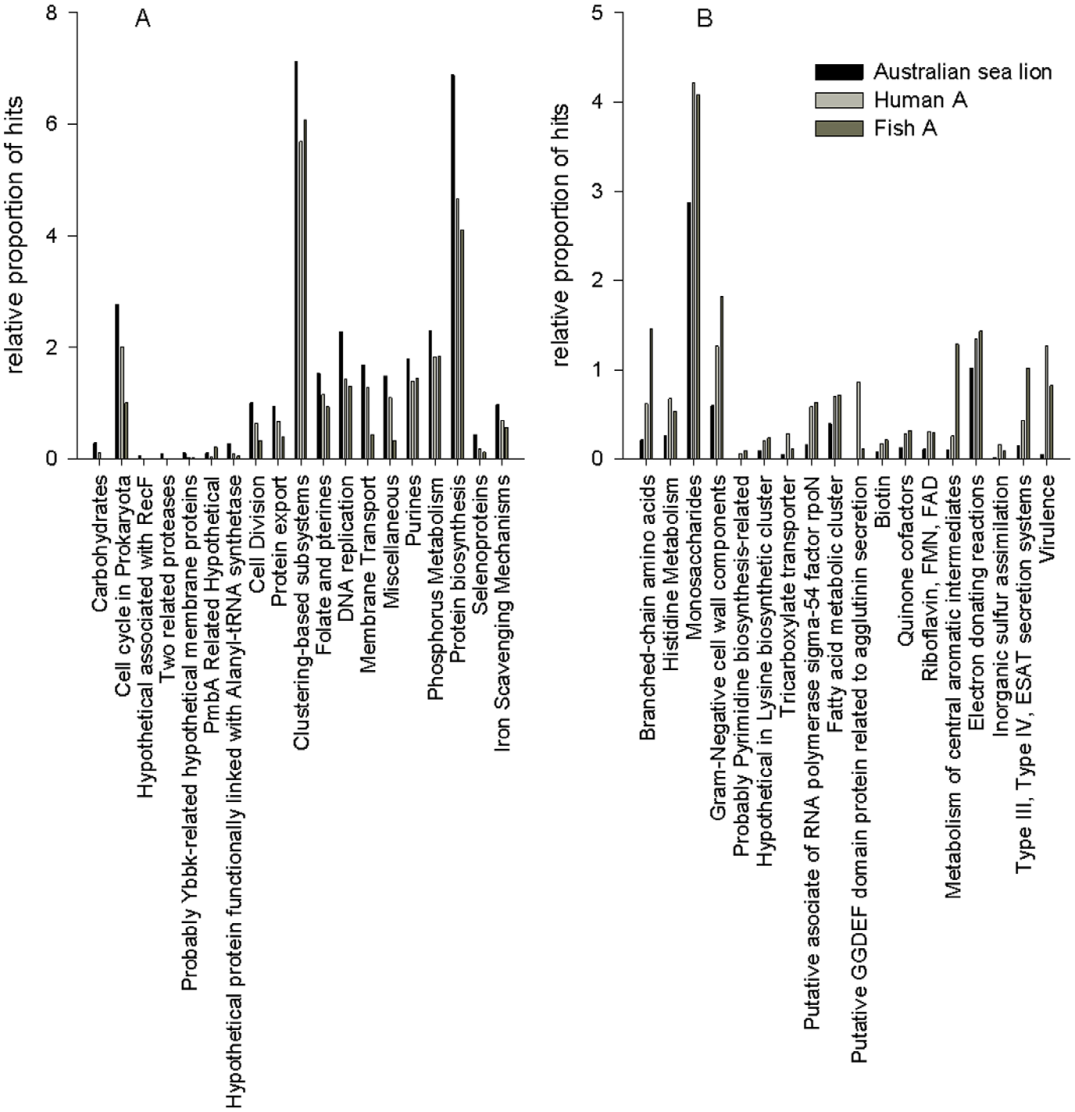
Metabolic Subsystems Over-represented and Under-represented in the Australian Sea Lion Faecal Microbiome compared to both Human A and Fish A Gut Microbiomes. A: The metabolic subsystems that are over-represented in the Australian sea lion faecal microbiome compared to Human A and Fish A gut microbiomes. B: The metabolic subsystems that are under-represented in the Australian sea lion faecal microbiome compared to Human A and Fish A gut microbiomes.

As a mammal that forages exclusively in the ocean, sea lions have a distinctive life history. Therefore, we also examined differences in the taxonomic and metabolic potential within the context of an ocean habitat, i.e., between the Australian sea lion faecal microbiome and an aquacultured fish (Fish A) gut microbiome. There was greater dissimilarity between the Australian sea lion faecal microbiomes and the fish faecal microbiome than was observed between the sea lion and human gut microbiomes (Figure 3). Fisher’s exact test revealed 35 significant differences in phyla between the Australian sea lion faecal microbiome and the Fish A faecal microbiome (Figure S3). The Australian sea lion faecal microbiome was over-represented in genes coding for *Firmicutes* and under-represented in genes coding for *Proteobacteria*. In regard to metabolic potential, the Australian sea lion microbiome was over-represented in comparison to Fish A microbiome in regard to 57 pathways and functions (Figure S4) including di- and oligosaccharides, cell cycle in prokaryota, DNA metabolism, membrane transport, protein biosynthesis, iron scavenging mechanisms and phosphorus metabolism. An analysis of the comparisons between the Australian sea lion microbiome and the human and fish microbiomes, reveals 19 metabolic processes in which the sea lion faecal microbiome is significantly enriched in comparison to both Human A and Fish A microbiomes (Figure 4A) and 18 processes in which both the Human A and Fish A microbiomes are significant enriched in comparison to the Australian sea lion faecal microbiome (Figure 4B).

**Figure 5:**
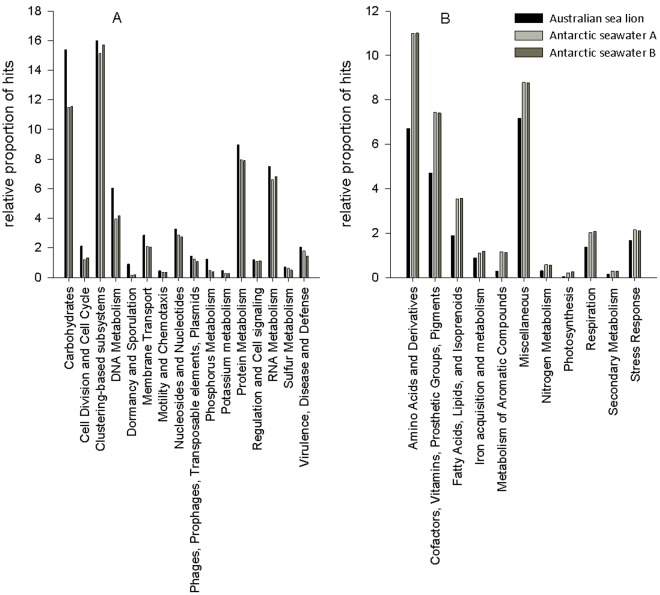
Metabolic Subsystems Over-represented and Under-represented in the Australian Sea Lion Faecal Microbiome compared to two Antarctic Seawater Microbiomes. A: The metabolic subsystems that are over-represented in the Australian sea lion faecal microbiome compared to two Antarctic seawater microbiomes. B: The metabolic subsystems that are under-represented in the Australian sea lion faecal microbiome compared to two Antarctic seawater microbiomes.

To further examine differences in metabolic potential within the context of an ocean habitat, we compared the metabolic potential of the Australian sea lion faecal microbiome to two Antarctic seawater microbiomes (termed Antarctic seawater A and Antarctic seawater B). Fisher’s exact test revealed 28 significant differences in metabolic potential between the Australian sea lion microbiome and Antarctic seawater sample A (Figure S5) and 27 significant differences in metabolic potential between the Australian sea lion faecal microbiome and Antarctic seawater sample B (Figure S6). There were 16 metabolic processes that were over-represented in the Australian sea lion faecal microbiome compared to both Antarctic seawater samples (Figure 5A) and 11 metabolic processes that were under-represented in the Australian sea lion faecal microbiome compared to both Antarctic seawater samples (Figure 5B).

## Discussion

### Australian Sea Lion Gut Microbiome Taxonomy

Our findings indicate that the Australian sea lion gut microbiome is dominated by the same four bacterial phyla that dominate the human gut (*Firmicutes*, *Proteobacteria, Bacteroidetes, Actinobacteria*) [12]. Compared to both Human A and Fish A faecal microbiomes, the Australian sea lion microbiome was over-represented in *Firmicutes* (Figures S1 and S3). In humans and mice, the relative proportion of *Firmicutes* to *Bacteriodetes* has been found to be a factor in obesity, with obese humans and mice having relatively fewer *Bacteriodetes* and more *Firmicutes* compared to lean subjects [13]–[15]. In the Australian sea lion faecal microbiome, the percentage of *Firmicutes* (80% of total sequences) to *Bacteriodetes* (2%) is similar to the relative proportions in obese mice and human subjects [13]. While many factors, such as diet and physiology, may influence body mass, the faecal microbiome of the Australian sea lion may confer a predisposition towards excess body fat. Excess body fat is an advantage for an endothermic mammal such as a sea lion that must maintain a stable, high (36 to 38°C) body temperature despite living in a fluid in which heat is conducted away from the body at 25 times faster than in air [16].

### Australian Sea Lion Microbiome Metabolic Potential

As in other gut microbiomes core metabolic functions including carbohydrate and protein metabolism dominated the Australian sea lion gut microbiome [5]. Carbohydrates serve an important role in energy storage within the gut. Protein metabolism is also a core function of the gut microbiome. While most microorganisms and plants can biosynthesise amino acids, animals must consume proteins as part of their diet in order to gain the amino acids needed for cell functioning. There were 63 significant differences in metabolic potential between Australian sea lion and Human A microbiomes (Figure S2) and 110 significant differences observed between the Australian sea lion and Fish A microbiomes (Figure S4). Overall, 19 metabolic processes were significantly enriched in the Australian sea lion microbiome compared to both the Human A and Fish A microbiomes (Figure 4A).

The Australian sea lion gut microbiome had significantly enriched numbers of genes coding for protein biosynthesis and membrane transport. Membrane transport genes are often overrepresented in gut microbiomes [17]. Diets high in fish have high levels of purines [18] and the high purine levels in the exclusive fish and cephalopod diet of Australian sea lions [19] may provide the resources for the observed over-representation of genes associated with DNA replication, DNA repair and cell division in the sea lion gut microbiome. Selenoproteins were also enhanced in the Australian sea lion microbiome compared to Human A and Fish A microbiomes which may suggest that the Australian sea lion gut is nutrient deficient relative to other gut microbiomes. Selenoproteins are involved in glycine reductase activity which incorporates the use of dithiol to reduce glycine to acetate and ammonia [20]. Glycine reductase activity is increased when *Clostridia* are grown in nutrient deficient conditions [21].

Further evidence for nutrient limitation within the Australian sea lion gut, specific to life in an ocean environment, is found in the over-representation of phosphorus metabolism and iron scavenging mechanism genes compared to both Fish A and Human A microbiomes. Iron is the limiting nutrient for many open ocean ecosystems [22] and increased iron uptake ability and phosphorus metabolism potential may allow for marine organisms to survive in ecosystems low in these essential nutrients. Foraging in the nutrient poor open ocean may have influenced the metabolism of the Australian sea lion gut microbiome in such a way as to ensure maximum uptake and metabolism of the limiting and valuable nutrients necessary for growth and reproduction.

Compared to the Antarctic seawater microbiomes, the Australian sea lion faecal microbiome was over-represented in 16 processes including phosphorus metabolism, potassium metabolism, sulphur metabolism and genes involved in virulence, disease and defence. Similar to comparisons with Human A and Fish A microbiomes, the Australian sea lion faecal microbiome was again over-represented in genes coding for membrane transport, cell division and carbohydrate metabolism (Figure 5A). The Australian sea lion faecal microbiome was under-represented in 11 metabolic processes including iron acquisition and metabolism, nitrogen metabolism, photosynthesis, respiration and metabolism of aromatic compounds when compared to both Antarctic seawater samples (Figure 5B).

### Environmental Consequences of Australian Sea Lion Defecations

The enriched number of genes coding for phosphorus metabolism in Australian sea lion faeces compared to Human A, Fish A and Antarctic seawater samples may have important environmental consequences if the sea lion faeces is defecated into surface waters. Bacteria require carbon, phosphorus, nitrogen and micronutrients including iron for growth and are net consumers of these nutrients in energy-poor environments. However, in nutrient-rich environments like the surface of a faecal particle, bacteria can solubilise more Fe, P and N from faecal matter than they require for their own growth (uncoupled solubilisation) [23]. This leads to leaching of these nutrients into the surrounding waters [23] where they can become available for free living microbes. Therefore, the bacteria in Australian sea lion faeces may limit nutrient sinkage to depth and enhance the persistence of nutrients in the photic zone where they are available to support primary production by phytoplankton [24].

### Conclusion

This metagenomic analysis reveals the genetic content and metabolic potential of an Australian sea lion gut microbiome. The phylogeny of the Australian sea lion gut microbiome is characterised by a high *Firmicutes* to *Bacteriodetes* ratio, which indicates a predisposition towards excess body fat in other mammals. The metabolic potential of the Australian sea lion gut microbiome was more similar to human gut microbiomes than cow gut, chicken cecum, fish guts, seawater samples or whale fall microbiomes. Compared to a human gut microbiome, the Australian sea lion gut microbiome had enriched numbers of genes coding for iron scavenging mechanisms and phosphorus metabolism. This finding suggests that Australian sea lion faeces contains bacteria able to assimilate and metabolize nutrients and is an important addition to the developing research showing that marine mammal faeces contribute to ocean nutrient dynamics.

## Supporting Information

Figure S1 **Statistical Differences in Taxonomic Diversity between Australian Sea Lion and Human A Faecal Microbiomes.** Symbols to the right are metabolic subsystems that are over-represented in the Australian sea lion (•) faecal microbiome compared to the Human A faecal microbiome. Symbols to the left are over-represented in the Human A (•) faecal microbiome compared to the Australian sea lion faecal microbiome.
(PDF)

Figure S2 **Statistical Differences in Metabolic Potential between the Australian Sea Lion and Human A Faecal Microbiomes.** Symbols to the right are metabolic subsystems that are over-represented in the Australian sea lion (•) faecal microbiome compared to the Human A faecal microbiome. Symbols to the left are over-represented in the Human A (•) faecal microbiome compared to the Australian sea lion faecal microbiome.
(PDF)

Figure S3 **Statistical Differences in Taxonomic Diversity between Australian Sea Lion and Fish A Faecal Microbiomes.** Symbols to the right are metabolic subsystems that are over-represented in the Australian sea lion (•) faecal microbiome compared to the Fish A faecal microbiome. Symbols to the left are the metabolic subsystems over-represented in the Fish A (•) faecal microbiome compared to the Australian sea lion faecalmicrobiome.
(PDF)

Figure S4 **Statistical Differences in Metabolic Potential between the Australian Sea Lion and Fish A Faecal Microbiomes.** Symbols to the right are metabolic subsystems that are over-represented in the Australian sea lion (•) faecal microbiome compared to the Fish A faecal microbiome. Symbols to the left are over-represented in the Fish A (•) faecal microbiome compared to the Australian sea lion faecal microbiome.
(PDF)

Figure S5 **Statistical Differences in Metabolic Potential between the Australian Sea Lion and Antarctic Seawater A Microbiomes.** Symbols to the right are metabolic subsystems that are over-represented in the Australian sea lion (•) faecal microbiome compared to the Antarctic Seawater A microbiome. Symbols to the left are over-represented in the Antarctic Seawater A (•)microbiome compared to the Australian sea lion faecal microbiome.
(PDF)

Figure S6 **Figure S6. Statistical Differences in Metabolic Potential between the Australian Sea Lion and Antarctic Seawater B Microbiomes.** Symbols to the right are metabolic subsystems that are over-represented in the Australian sea lion (•) faecal microbiome compared to the Antarctic Seawater B microbiome. Symbols to the left are over-represented in the Antarctic Seawater B (•)microbiome compared to the Australian sea lion faecal microbiome.
(PDF)

## References

[1] Gill SR, Pop M, DeBoy RT, Eckburg PB, Turnbaugh PJ (2006). Metagenomic Analysis of the Human Distal Gut Microbiome.. Science.

[2] Bäckhed F, Ley RE, Sonnenburg JL, Peterson DA, Gordon JI (2005). Host-Bacterial Mutualism in the Human Intestine.. Science.

[3] Wikoff WR, Anfora AT, Liu J, Schultz PG, Lesley SA (2009). Metabolomics analysis reveals large effects of gut microflora on mammalian blood metabolites.. Proc Natl Acad Sci.

[4] Turnbaugh PJ, Ridaura VK, Faith JJ, Rey FE, Knight R (2009). The Effect of Diet on the Human Gut Microbiome: A Metagenomic Analysis in Humanized Gnotobiotic Mice.. Sci Translatl Med.

[5] Qin J, Ruiqiang L, Raes J, Arumugam M, Burgdorf KS (2010). A human gut microbial gene catalogue established by metagenomic sequencing.. Nature.

[6] Lavery TJ, Roudnew B, Gill P, Seymour J, Seuront L (2010). Iron defecation by sperm whales stimulates carbon export in the Southern Ocean.. Proc Royal Soc B: Biol Sci.

[7] Goldsworthy SD, Hamer D, Page B (2007). Assessment of the implications of interactions between fur seals and sealions and the southern rock lobster and gillnet sector of the Southern and Eastern scalefish and shark fishery (SESSF) in South Australia..

[8] Costa DP, Gales NJ (2003). Energetics of a benthic diver: Seasonal foraging ecology of the Australian sea lion, Neophoca cinerea.. Ecol Monographs.

[9] Meyer F, Paarmann D, D’Souza M, Olson R, Glass EM (2008). The metagenomics RAST server - a public resource for the automatic phylogenetic and functional analysis of metagenomes.. BMC Bioinformatics.

[10] Overbeek R, Begley T, Butler RM, Choudhuri JV, Chuang H-Y (2005). The Subsystems Approach to Genome Annotation and its Use in the Project to Annotate 1000 Genomes.. Nucleic Acids Res.

[11] Parks DH, Beiko G (2010). Identifying biologically relevant differences between metagenomic communities.. Bioinformatics.

[12] Tasse L, Bercovici J, Pizzut-Serin S, Robe P, Tap J (2010). Functional metagenomics to mine the human gut microbiome for dietary fiber catabolic enzymes.. Genome Research.

[13] Ley RE, Bäckhed F, Turnbaugh P, Lozupone CA, Knight RD (2005). Obesity alters gut microbial ecology.. Proc Natl Acad Sci.

[14] Ley RE, Turnbaugh PJ, Klein S, Gordon JI (2006). Microbial ecology: Human gut microbes associated with obesity.. Nature.

[15] Turnbaugh PJ, Ley RE, Mahowald MA, Magrini V, Mardis ER (2006). An obesity-associated gut microbiome with increased capacity for energy harvest.. Nature.

[16] Pabst DA, Rommel SA, McLellan WA (1999). The functional morphology of marine mammals, in Biology of marine mammals, J.E. Reynolds III and S. Rommel, Editors..

[17] Xu J, Mahowald MA, Ley RE, Lozupone CA, Hamady M (2007). Evolution of Symbiotic Bacteria in the Distal Human Intestine.. PLoS Biol.

[18] Choi HK, Atkinson K, Karlson EW, Willett W, Curhan G (2004). Purine-Rich Foods, Dairy and Protein Intake, and the Risk of Gout in Men.. New England J Med.

[19] Gales NJ, Cheal AJ (1992). Estimating diet composition of the Australian sea lion (Neophoa cinerea) from scat analysis: an unrliable technique.. Wildlife Res.

[20] Tanaka H, Stadtman TC (1979). Selenium-dependent Clostridial glycine reductase.. The J Biol Chem.

[21] Venugopalan V (1980). Influence of growth conditions on glycine reductase of Clostridium sporogenes.. J Bacteriol.

[22] Martin JH, Coale KH, Johnson KS, Fitzwater SE, Gordon RM (1994). Testing the iron hypothesis in ecosystems of the equatorial Pacific Ocean.. Nature.

[23] Azam F, Smith DC, Steward GF, Hagstrom A (1994). Bacteria-organic matter coupling and its significance for oceanic carbon cycling.. Microb Ecol.

[24] Azam F, Malfatti F (2007). Microbial structuring of marine ecosystems.. Nature Rev Microbiol.

