# Supplementary figures and images for: High Nutrient Transport and Cycling Potential Revealed in the Microbial Metagenome of Australian Sea Lion (Neophoca cinerea) Faeces
Source: PLoS One. 2012 May 11;7(5):e36478. doi: 10.1371/journal.pone.0036478 (PMC3350522; doi:10.1371/journal.pone.0036478)

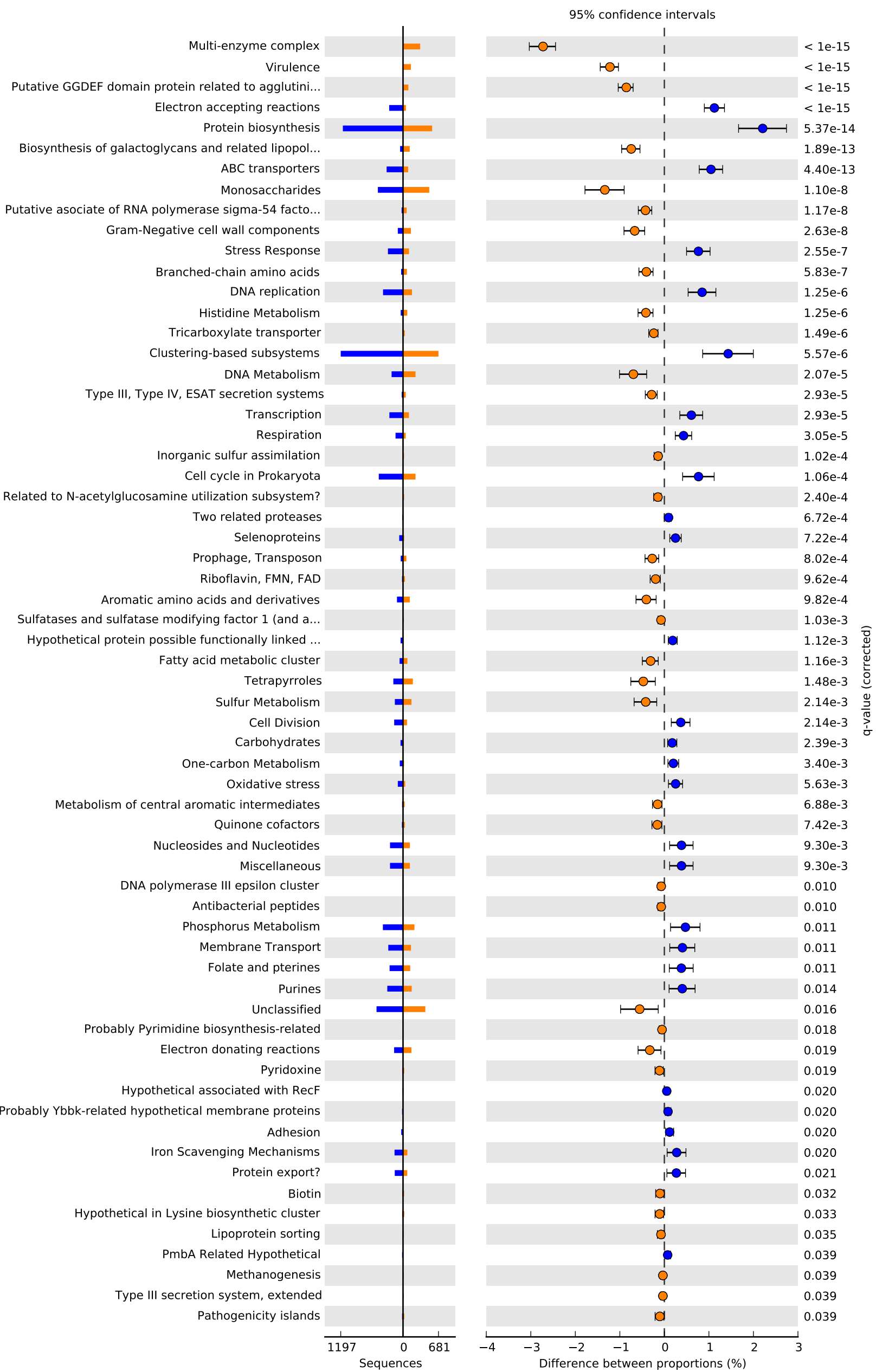

Supplement: Figure S1 — Statistical Differences in Taxonomic Diversity between Australian Sea Lion and Human A Faecal Microbiomes. Symbols to the right are metabolic subsystems that are over-represented in the Australian sea lion (•) faecal microbiome compared to the Human A faecal microbiome. Symbols to the left are over-represented in the Human A (•) faecal microbiome compared to the Australian sea lion faecal microbiome. (PDF) [file pone.0036478.s001.pdf]

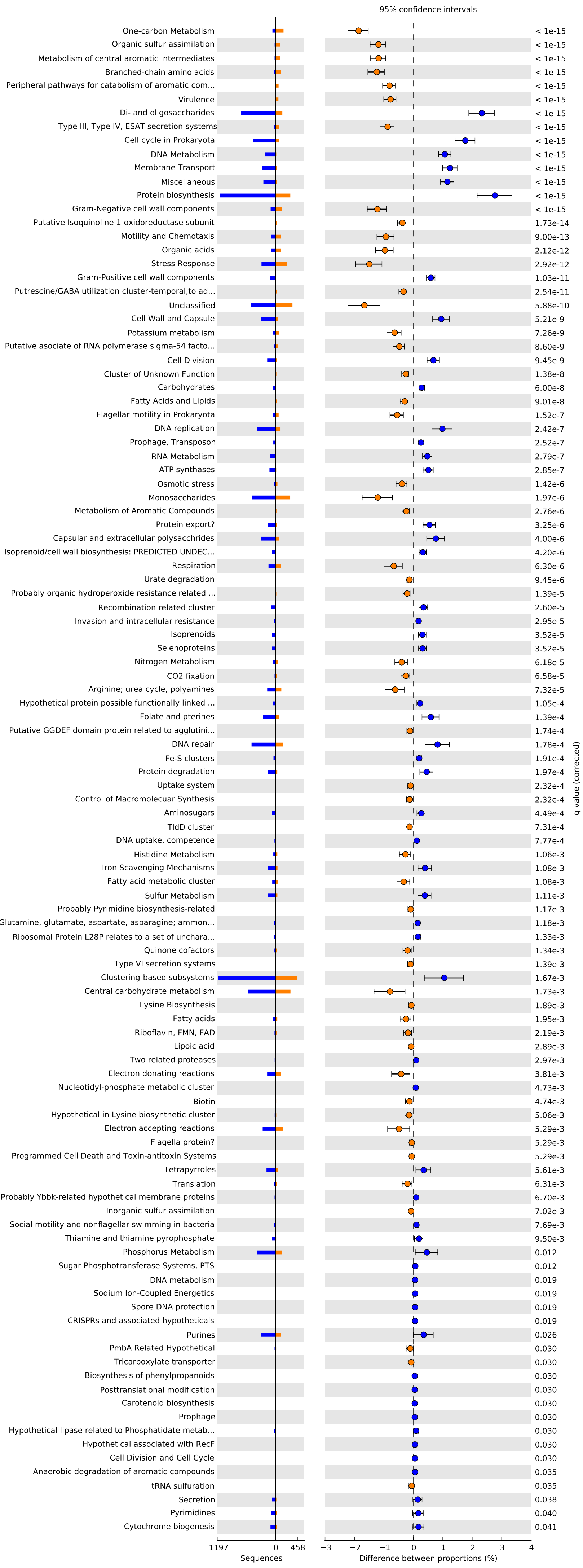

Supplement: Figure S2 — Statistical Differences in Metabolic Potential between the Australian Sea Lion and Human A Faecal Microbiomes. Symbols to the right are metabolic subsystems that are over-represented in the Australian sea lion (•) faecal microbiome compared to the Human A faecal microbiome. Symbols to the left are over-represented in the Human A (•) faecal microbiome compared to the Australian sea lion faecal microbiome. (PDF) [file pone.0036478.s002.pdf]

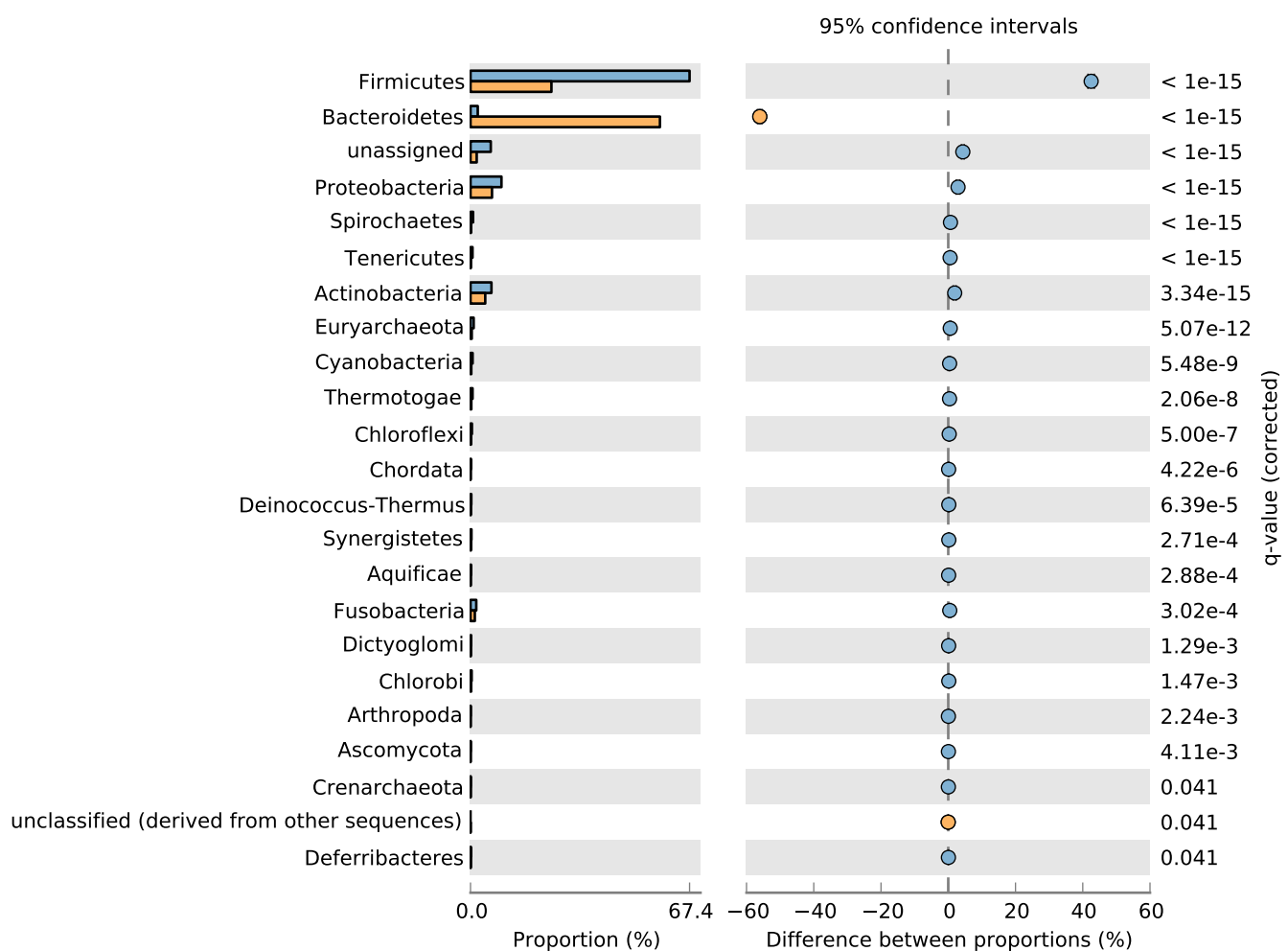

Supplement: Figure S3 — Statistical Differences in Taxonomic Diversity between Australian Sea Lion and Fish A Faecal Microbiomes. Symbols to the right are metabolic subsystems that are over-represented in the Australian sea lion (•) faecal microbiome compared to the Fish A faecal microbiome. Symbols to the left are the metabolic subsystems over-represented in the Fish A (•) faecal microbiome compared to the Australian sea lion faecalmicrobiome. (PDF) [file pone.0036478.s003.pdf]

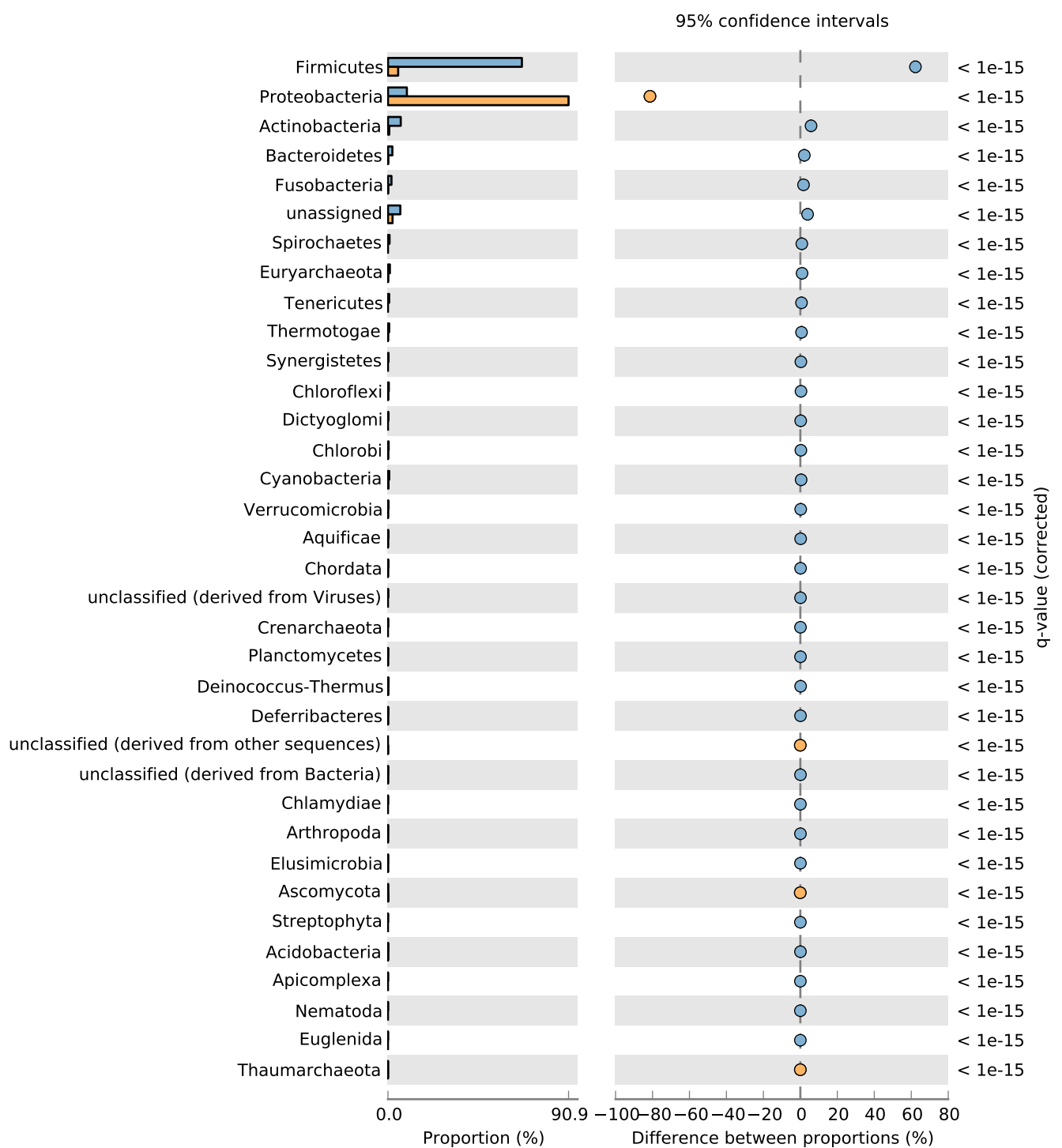

Supplement: Figure S4 — Statistical Differences in Metabolic Potential between the Australian Sea Lion and Fish A Faecal Microbiomes. Symbols to the right are metabolic subsystems that are over-represented in the Australian sea lion (•) faecal microbiome compared to the Fish A faecal microbiome. Symbols to the left are over-represented in the Fish A (•) faecal microbiome compared to the Australian sea lion faecal microbiome. (PDF) [file pone.0036478.s004.pdf]

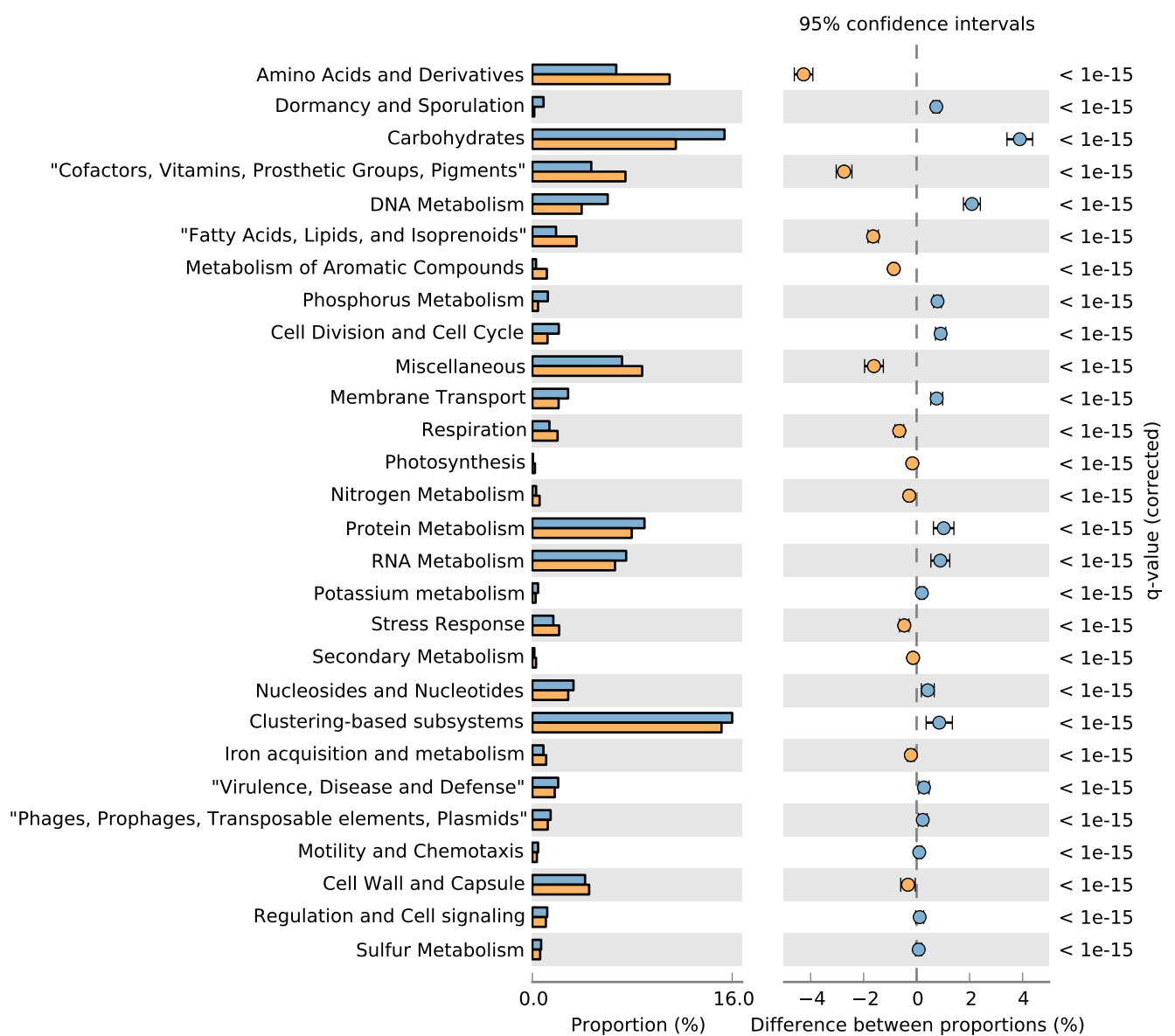

Supplement: Figure S5 — Statistical Differences in Metabolic Potential between the Australian Sea Lion and Antarctic Seawater A Microbiomes. Symbols to the right are metabolic subsystems that are over-represented in the Australian sea lion (•) faecal microbiome compared to the Antarctic Seawater A microbiome. Symbols to the left are over-represented in the Antarctic Seawater A (•)microbiome compared to the Australian sea lion faecal microbiome. (PDF) [file pone.0036478.s005.pdf]

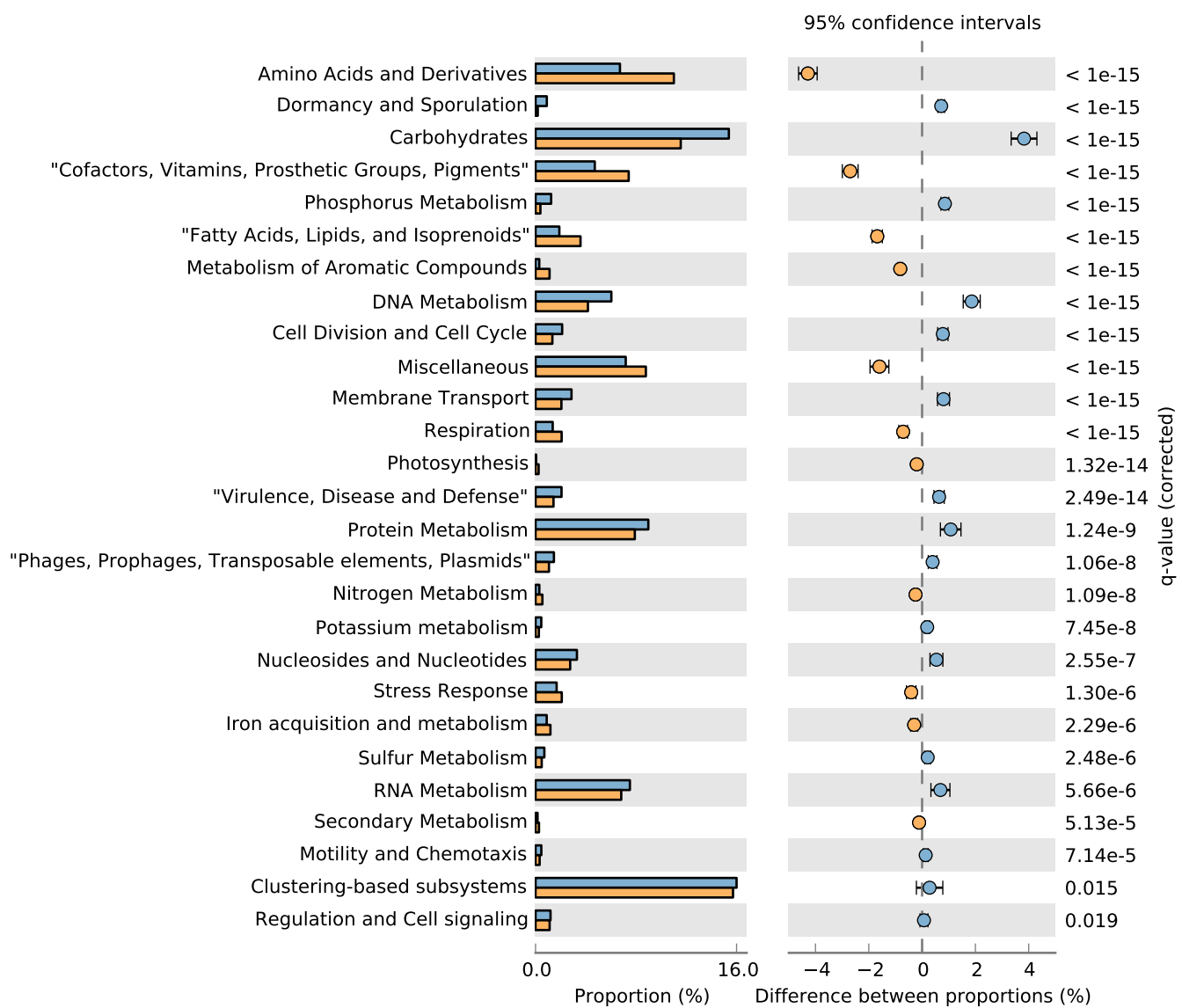

Supplement: Figure S6 — Statistical Differences in Metabolic Potential between the Australian Sea Lion and Antarctic Seawater B Microbiomes. Symbols to the right are metabolic subsystems that are over-represented in the Australian sea lion (•) faecal microbiome compared to the Antarctic Seawater B microbiome. Symbols to the left are over-represented in the Antarctic Seawater B (•)microbiome compared to the Australian sea lion faecal microbiome. (PDF) [file pone.0036478.s006.pdf]
